# Chinks in the Armor? Filaggrin-Depleted Skin Could Increase Environmental Exposures

**DOI:** 10.1289/ehp.122-A108

**Published:** 2014-04-01

**Authors:** Lindsey Konkel

**Affiliations:** Lindsey Konkel is a Worcester, MA–based journalist who reports on science, health, and the environment. She is an editor for *Environmental Health News* and *The Daily Climate*.

Young, healthy men with a gene variant that may impair skin barrier function had higher urine levels of certain phthalates than men without the variant, according to a group of Danish researchers.^1^ The findings, reported in this issue of *EHP*, suggest that a genetic factor may determine the extent of a person’s exposure to an environmental chemical.

“This idea that a certain level of exposure to a chemical can mean something very different depending on your genetic makeup is quite new and important,” says Shanna Swan, a reproductive health scientist at the Mount Sinai School of Medicine in New York, who was not involved in the study.

**Figure:**
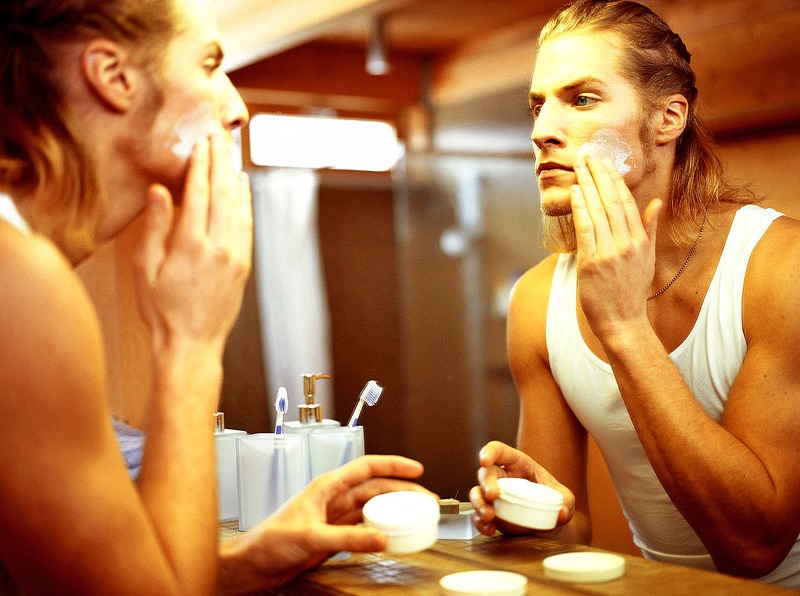
Men with a gene variant that predisposed them to dry skin were more likely to have higher urinary concentrations of phthalates found in skin care products. © Anna Peisl/Corbis

Small percentages of Europeans and Asians have a genetic mutation that diminishes the production of filaggrin,^2^ an epidermal protein that helps to lock moisture into the skin and keep out pathogens, allergens, and chemicals. People with filaggrin-depleted skin may be more likely to have allergic skin disorders.^3^ The researchers hypothesized that these people may also absorb phthalates through the skin at higher rates than people with normal filaggrin production.

Certain phthalates—a large group of chemicals found in cosmetics, fragrances, solvents, and plastics—have been associated with markers of decreased testicular function in some human studies^4^ and altered male genital development in animal research.^5^ They’ve also been associated with diabetes,^6^ asthma,^7^ attention disorders,^8^ and obesity^9^ in some epidemiological studies.

In the current study, researchers measured levels of phthalate metabolites in the urine of 861 Danish men between the ages of 18 and 22. Sixty-five men, roughly 7.5% of the study participants, carried at least one nonfunctional copy of the filaggrin gene (*FLG*), indicating diminished filaggrin production. On average, those men had 33% higher urinary concentrations of a metabolite of di-*n-*butyl phthalate (DnBP) than men with two functional *FLG* copies. They also higher average levels of metabolites of diisobutyl phthalate (DiBP) and butylbenzyl phthalate (BBzP). These low-molecular-weight phthalates are commonly found in cosmetics and personal care products.

However, the average urinary concentration of a metabolite for diethyl phthalate (DEP), the most frequently detected phthalate in personal care products, was not significantly higher in men with *FLG* mutant alleles. Metabolites of several high-molecular-weight phthalates, which are primarily found in PVC plastics, also were not significantly higher in this group.

When the researchers compared concentrations of reproductive hormones and markers of semen quality between the two groups of men, they found no significant differences.

It’s unclear from the study why men with *FLG* mutations tended to have higher levels of some phthalates. It’s possible their skin was more permeable to chemicals they encountered. People with reduced filaggrin production also tend to have drier skin, says lead study author Ulla Nordström Joensen, a urologist at Copenhagen University Hospital, so it’s plausible that higher phthalate levels could be the result of this group using more skin care products. “Whatever the explanation, they appear to be more exposed, which is potentially concerning,” Joensen says.

In 2008 the U.S. federal government heavily restricted the use of DBP, BBzP, and di(2-ethylhexyl) phthalate in toys and other children’s products, while the European Union has placed similar restrictions on these chemicals.^10^ For the general population, exposure to high-molecular-weight phthalates appears to result largely from diet, whereas nondietary routes such as use of personal care products and indoor dust and air appear to explain most exposure to low-molecular-weight phthalates.^11^

Future studies should investigate whether similar associations appear between *FLG* mutations and phthalate exposures in other population groups, such as women and children, and whether *FLG* mutation carriers have greater exposure to other environmental chemicals. If it turns out these individuals’ skin is more permeable to phthalates, Joensen says, it’s possible they should pay special attention to dermal chemical exposures in general.

## References

[1] Joensen UN Associations of filaggrin gene loss-of-function variants with urinary phthalate metabolites and testicular function in young Danish men. Environ Health Perspect 122 3 345 350 2014; 10.1289/ehp.1306720 24380925 PMC3984221

[2] Irvine AD Filaggrin mutations associated with skin and allergic diseases. N Engl J Med 365 14 1315 1327 2011; 10.1056/NEJMra1011040 21991953

[3] Scharschmidt TC Filaggrin deficiency confers a paracellular barrier abnormality that reduces inflammatory thresholds to irritants and haptens. J Allergy Clin Immunol 124 3 496 506.e6 2009; 10.1016/j.jaci.2009.06.046 19733297 PMC2881668

[4] Mendiola J Urinary concentrations of di(2-ethylhexyl) phthalate metabolites and serum reproductive hormones: pooled analysis of fertile and infertile men. J Androl 33 3 488 498 2012; 10.2164/jandrol.111.013557 21597090 PMC3433231

[5] Swan SH Decrease in anogenital distance among male infants with prenatal phthalate exposure. Environ Health Perspect 113 8 1056 1061 2005; 10.1289/ehp.8100 16079079 PMC1280349

[6] James-Todd T Urinary phthalate metabolite concentrations and diabetes among women in the National Health and Nutrition Examination Survey (NHANES) 2001–2008. Environ Health Perspect 120 9 1307 1313 2012; 10.1289/ehp.1104717 22796563 PMC3440117

[7] Bertelsen RJ Urinary biomarkers for phthalates associated with asthma in Norwegian children. Environ Health Perspect 121 2 251 256 2013; 10.1289/ehp.1205256 23164678 PMC3569683

[8] Chopra V Association between phthalates and attention deficit disorder and learning disability in U.S. children, 6–15 years. Environ Res 128 64 69 2014; 10.1016/j.envres.2013.10.004 24267794 PMC3889659

[9] Trasande L Race/ethnicity-specific associations of urinary phthalates with childhood body mass in a nationally representative sample. Environ Health Perspect 121 4 501 506 2013; 10.1289/ehp.1205526 23428635 PMC3620751

[10] Zota AR Temporal trends in phthalate exposures: findings from the National Health and Nutrition Examination Survey, 2001–2010. Environ Health Perspect 122 3 235 241 2014; 10.1289/ehp.1306681 24425099 PMC3948032

[11] Koch HM Identifying sources of phthalate exposure with human biomonitoring: results of a 48 h fasting study with urine collection and personal activity patterns. Int J Hyg Environ Health 216 6 672 681 2013; 10.1016/j.ijheh.2012.12.002 23333758

